# Activation of Polyamine Catabolism by N^1^,N^11^-Diethylnorspermine in Hepatic HepaRG Cells Induces Dedifferentiation and Mesenchymal-Like Phenotype

**DOI:** 10.3390/cells7120275

**Published:** 2018-12-18

**Authors:** Olga N. Ivanova, Anastasiya V. Snezhkina, George S. Krasnov, Vladimir T. Valuev-Elliston, Olga A. Khomich, Alexey R. Khomutov, Tuomo A. Keinanen, Leena Alhonen, Birke Bartosch, Anna V. Kudryavtseva, Sergey N. Kochetkov, Alexander V. Ivanov

**Affiliations:** 1 Engelhardt Institute of Molecular Biology, Russian Academy of Sciences, 119991 Moscow, Russia; olgaum@yandex.ru (O.N.I.); leftger@rambler.ru (A.V.S.); gskrasnov@mail.ru (G.S.K.); gansfaust@mail.ru (V.T.V.-E.); oakhomich@gmail.com (O.A.K.); alexkhom@list.ru (A.R.K.); rhizamoeba@mail.ru (A.V.K.); kochet@eimb.ru (S.N.K.); 2 Cancer Research Center Lyon, INSERM U1052 and CNRS 5286, Lyon University, 69000 Lyon, France; birke.bartosch@inserm.fr; 3 School of Pharmacy, Biocenter Kuopio, University of Eastern Finland, FI-70211 Kuopio, Finland; tuomo.keinanen@uef.fi (T.A.K.); alhonenl@gmail.com (L.A.)

**Keywords:** polyamines, HepaRG, polyamine catabolism, dedifferentiation, EMT, spermidine, polyamine analogues, NextSeq, hepatocytes

## Abstract

Tumorigenesis is accompanied by the metabolic adaptation of cells to support enhanced proliferation rates and to optimize tumor persistence and amplification within the local microenvironment. In particular, cancer cells exhibit elevated levels of biogenic polyamines. Inhibitors of polyamine biosynthesis and inducers of their catabolism have been evaluated as antitumor drugs, however, their efficacy and safety remain controversial. Our goal was to investigate if drug-induced modulation of polyamine metabolism plays a role in dedifferentiation using differentiated human hepatocyte-like HepaRG cell cultures. N^1^,N^11^-diethylnorspermine (DENSpm), a potent inducer of polyamine catabolism, triggered an epithelial-mesenchymal transition (EMT)-like dedifferentiation in HepaRG cultures, as shown by down-regulation of mature hepatocytes markers and upregulation of classical EMT markers. Albeit the fact that polyamine catabolism produces H2O2, DENSpm-induced de-differentiation was not affected by antioxidants. Use of a metabolically stable spermidine analogue showed furthermore, that spermidine is a key regulator of hepatocyte differentiation. Comparative transcriptome analyses revealed, that the DENSpm-triggered dedifferentiation of HepaRG cells was accompanied by dramatic metabolic adaptations, exemplified by down-regulation of the genes of various metabolic pathways and up-regulation of the genes involved in signal transduction pathways. These results demonstrate that polyamine metabolism is tightly linked to EMT and differentiation of liver epithelial cells.

## 1. Introduction

Oncological malignancies comprise a vast group of diseases that tremendously shorten life length and quality. In recent decades significant progress was achieved in cancer diagnostics and treatment. Nevertheless, treatment efficacy of a number of cancers including stomach, brain, liver, lung, and pancreatic cancers is still not efficient, and survival rates of concerned patients are extremely low. Liver cancer is the fifth most common type of cancer in men and women combined, and the second most deadly cancer [1]. Its 5-year survival rate drops from 31% at early detection to 3% for patients in whom the cancer has spread to a distant part of the body [2]. The mechanisms of cell transformation in liver and other organs are not fully understood but it is clear that among the important processes are changes in various metabolic systems. Metabolic pathways are altered in cancer not only to sustain cell proliferation and biosynthesis but also for immunomodulation and symbiosis [3]. Many cancer cells are characterized by enhanced glycolysis and glutaminolysis to fuel production of ATP in the TCA cycle, as well as biosynthesis of nucleotides, lipids, NADPH, and other molecules essential for the rapid proliferation of tumor cells. However, additional metabolic pathways are frequently altered as well, including biogenic polyamines.

The polyamines spermine and spermidine as well as diamine putrescine are metabolites characterized by aliphatic oligocations and present in all cell types of the body at submillimolar and millimolar levels [4,5]. Being positively-charged compounds they interact with various nucleic acids, proteins and nucleoprotein complexes. Involvement of spermine and spermidine in regulation of various processes in the cells including DNA replication and transcription, RNA processing and translation, scavenging of reactive oxygen species (ROS) has been documented [4,6,7]. The levels of polyamines are tightly regulated by both biosynthesis and degradation in rate-limiting reactions that are controlled by several enzymes. These include ornithine decarboxylase (ODC), which catalyzes the conversion of ornithine into putrescine, spermidine/spermine-N^1^-acetyl transferase (SSAT) and spermine oxidase (SMO), both of which mediate alternative catabolic pathways. Acetylated spermine and spermidine, produced by SSAT, can either undergo rapid conversion into spermidine and putrescine by acetylpolyamine oxidase (APAO), or be excreted from the cell. To some extent, polyamine levels in cells are also dependent upon polyamine uptake through yet unidentified transporters.

It has been shown that spermine and spermidine levels are significantly increased in various types of cancers including liver, colon, and brain [8,9]. In contrast, a decrease of spermine and spermidine content in vitro by inhibition of their biosynthesis or activation of their degradation leads to the inhibition of cell growth or even cell death [8]. Therefore, polyamine depletion in vivo has been considered as a promising anticancer strategy. Indeed, inhibitors of ODC or inducers of SSAT, such as α-difluoromethylornithine (DFMO) and N^1^,N^11^-diethylnorspermine (DENSpm), were evaluated as anticancer agents both in laboratory model systems and in several clinical trials [10,11,12,13,14,15]. DFMO blocks polyamine biosynthesis by acting as an irreversible ODC inhibitor and therefore causes depletion of putrescine and spermidine [8]. DENSpm is a bis-alkylated polyamine analogue which is capable of inducing SSAT expression by up to 10–1000 fold thus leading to rapid exhaustion of spermine and spermidine levels and production of hazardous hydrogen peroxide and N-acetyl-3-aminopropanal [8,9]. DENSpm-treated cells exhibit slower proliferation rates or undergo apoptosis. In animal models DENSpm was shown to exhibit promising anticancer activity thus paving the way for clinical trials. However, in phase I trials for advanced hepatocellular carcinoma [10] and non-small cell lung carcinoma [11] or metastatic breast cancer [12], disease progression failed to stabilize in patients on DENSpm. Treatment of the patients with lung cancer showed minimal toxicity [11], whereas in other cases severe central neural system (CNS) toxicity was noted [13]. In addition, in clinical trials of liver cancer almost 30% of patients exhibited elevation of aspartate aminotransferase (AST), a marker for hepatotoxicity [10]. Currently, the efficacy of DENSpm and its possible adverse effects should be investigated in other pathologies. 

To date there are several pieces of evidence that show that polyamine depletion not only triggers cancer cell death but also affects pathway that trigger tissue fibrosis and cancer progression. Treatment of rat intestinal crypt cells with DFMO stimulates induction of the profibrotic transforming growth factor β1 (TGFβ1) at the posttranscriptional level, expression of its receptor and downstream Smad3/4 signaling [16,17,18]. DFMO-mediated decrease of polyamine content in these cells also decreased expression of the tight junction receptors occludin, claudins 2 and 3, and ZO-1 and -2 which are required for the formation and proper functioning of epithelia [19]. In canine kidney MDCK cells DFMO also resulted in a moderately enhanced TGFβ1 secretion, but no increased cell death was observed [20]. However, if combined with exogenous TGFβ1, DFMO augmented TGFβ1-induced epithelial to mesenchymal transition (EMT) [20,21]. EMT is a process during which epithelial cells loose cell-cell contacts and polarity, alter their metabolism and acquire motility and other characteristics of mesenchymal cells [22]. In adults, EMT accounts for transformation of epithelial cells into myofibroblasts during fibrogenesis (type II EMT) and for progression from a primary carcinoma phenotype towards a metastatic phenotype (type III EMT).

The goal of our study was to explore whether interference with the metabolism of biogenic polyamines can induce EMT using the immortalized non-transformed liver cell line HepaRG. HepaRG cells were obtained from liver resection from a patient with hepatitis C virus-associated hepatocellular carcinoma [23]. Unlike hepatoma cell lines, the HepaRG cells are negative for α-fetoprotein and are not capable of forming tumors upon injection into mice [23,24]. HepaRG are hepatic bipotent progenitor cells that are capable of differentiation into a mixed culture of hepatocyte- and cholangiocyte-like cells whose properties are close to those of primary hepatocytes [23,25]. We show that DENSpm-augments catabolism of spermine and spermidine and induces an EMT-like dedifferentiation in a differentiated HepaRG culture. De-differentiation could be prevented by replenishment of polyamines in the form of metabolically-stable analogues. These data indicate that the polyamine metabolism and in particular levels of spermidine control differentiation of liver cells.

## 2. Materials and Methods

### 2.1. Materials

Fetal bovine serum (FBS), Williams E medium, dimethylsulfoxide (DMSO), bovine insulin, hydrocortisone hemisulfate, and MDL72.527 were obtained from Sigma (St. Louis, MO, USA), whereas MTT was purchased from Applichem (Darmstadt, Germany). DMEM was from Life Technologies (Carlsbad, CA, USA). Mint reverse transcriptase, qPCRmix-HS and qPCRmix-HS SYBR master mixes were purchased from Evrogen (Moscow, Russia). Unmodified DNA oligonucleotides were synthesized by Lytech (Moscow, Russia) or Evrogen, and Taqman probe was from DNA synthesis (Moscow, Russia). Primary rabbit antibodies to E-cadherin (24E10), N-cadherin (D4R1H), claudin-1 (D5H1D), and ZO-1 (D7D12) were purchased from Cell Signaling (Leiden, Netherlands), whereas primary mouse antibodies to β-actin (ab3280) and anti-rabbit (ab6721) and anti-mouse (ab6728) secondary antibodies conjugated to horseradish peroxidase (HRP) were from Abcam (Cambridge, UK). (*R*)-3-Methylspermidine (MeSpd) [26] and (*R*,*R*)-1,12-dimethylspermine (Me_2_Spm) [27] were synthesized as described earlier. DENSpm was earlier synthesized as described in [28]. DFMO was a kind gift of Prof. P. Woster (Medical University of South Carolina, Charleston, SC, USA).

### 2.2. Cell culture

Huh7.5 cells were obtained from Apath L.L.C. SkHep1 and HepG2 cells were from ATCC collection, HepaRG cells were previously described [23]. All cells except HepaRG were cultured in DMEM supplemented with 10% fetal bovine serum, and 2 mM glutamine and collected at 80–90% confluency. HepaRG cells were cultured in Williams E medium supplemented with 10% fetal bovine serum, 5 μg/mL insulin, 50 μM hydrocortisone, 50 U/mL penicillin, and 50 μg/mL streptomycin. Undifferentiated cells were collected and split at 80–90% confluency. Differentiation of HepaRG cells was achieved by cultivation of the cells at 100% confluency for two weeks in complemented Williams E medium followed by two weeks in the same medium supplemented with 1.8% DMSO, with changing medium every three days. To modulate the polyamine metabolism, cells were treated with 5 mM DFMO, 10 μM DENSpm or MDL72.527 alone or in the presence of 2.5 mM NAC, 100 μM trolox, 100 μM (*R*)-3-methylspermidine or 100 μM (*R*,*R*)-1,12-dimethylspermine. Cells were collected by scraping and split into three and stored at −80 °C for subsequent analysis by qPCR, Western blotting, or polyamine quantification.

### 2.3. Measurement of Polyamine Levels

For polyamine determination, cells from a 10 cm culture dish were collected by scraping, incubated in 300 μL of lysis buffer A (25 mM Tris-HCl, pH 7.4, 1 mM EDTA, 1% (*v*/*v*) Triton X-100) supplemented with protease inhibitor cocktail for 30 min on ice, and treated with sulfosalicylic acid to 5% final concentration. Precipitates, collected by centrifugation, were used for quantification of DNA levels by PicoGreen reagent (Invitrogen, Carlsbad, CA, USA) according to manufacturer’s instructions. The supernatants were supplemented with 1,7-diaminoheptane (internal control), and subjected to quantification of levels of individual polyamines as described previously [29]. The polyamine levels for each sample were normalized to levels of DNA.

### 2.4. Reverse-Transcription Polymerase Chain Reaction (RT-PCR)

Purification of total RNA and reverse transcription was carried out similarly to previously described [30]. QPCR was performed on a Lightcycler 96 system (Roche, Basel, Switzerland) using primers listed in the Table S1 by the SYBR Green approach in qPCRmix-HS SYBR mixture. Relative quantitative analysis was performed by comparing a threshold cycle number for the target genes and β-actin mRNA as a reference, amplified in separate tubes using primers and Taqman probes, respectively. A standard reaction mixture (25 µL) contained the respective primers, the Taqman probe (in case of β-actin), cDNA equivalent to 50 ng total RNA, and qPCRmix-HS SYBR+ROX or qPCRmix-HS master mix (Taqman protocol). The real-time PCR thermal conditions were 55 °C for 5 min, 95 °C for 10 min, followed by 40 cycles each at 95 °C for 10 s and 57 °C for 1 min (signal collection temperature).

### 2.5. Western Blot Analysis

The western blot analysis was performed according to the procedure described in [31]. Specifically, the membranes were incubated with the primary antibodies to E-cadherin (1:1000), N-cadherin (1:1000), ZO-1 (1:1000), claudin-1 (1:1000) or β-actin (0.2 μg/mL), followed by incubation with anti-rabbit (0.6 μg/mL) and anti-mouse (0.4 μg/mL) secondary HRP-conjugated antibodies. The quantification of protein levels was performed with TotalLab software (Totallab, Newcastle upon Tyne, UK).

### 2.6. Cell Viability Assay

The cells were grown on a 96-well plate, incubated in the presence of 1–40 mM DFMO, 3–200 μM DENSpm or 6–200 μM MDL72.527 for 72 h, then with 0.5 mg/mL MTT in the fresh medium for 4 h at 37 °C. Subsequently the medium was removed, the formazan crystals were dissolved in 2-propanol (100 μL/well) containing 0.04 M HCl, and optical density was measured at 544 nm on a Chameleon V microplate reader (Hydex Oy, Turku, Finland).

### 2.7. Isolation of RNA for NextSeq Sequencing

Total RNA was isolated from the cell lines using a MagNA Pure Compact RNA Isolation Kit (Roche, Basel, Switzerland) on a MagNA Pure Compact Instrument (Roche, Basel, Switzerland). The quality of isolated RNA was determined by an Agilent 2100 Bioanalyzer (Agilent Technologies, Santa Clara, USA). The RNA with RIN (RNA Integrity Number) values of more than eight were used only. Quantification of RNA was performed with a Qubit 2.0 Fluorometer (Thermo Fisher Scientific, Waltham, MA, USA).

### 2.8. cDNA Library Preparation and Sequencing

The cDNA libraries were prepared using a TruSeq RNA Library Preparation Kit v2 (Illumina, USA) according to the manufacturer’s instructions. Total RNA was purified using oligo-dT attached magnetic beads. Following purification, the mRNA was fragmented and then converted to double-stranded cDNA. The cDNA was subjected to ligation of the adaptors and amplification. The quality of final cDNA libraries was evaluated by qPCR on a Rotor-Gene Q Instrument (Qiagen, Hilden, Germany). Transcriptome sequencing was performed using NextSeq 500 System (Illumina) with 75 single end reads at the EIMB RAS “Genome” center (http://www.eimb.ru/rus/ckp/ccu_genome_c.php).

### 2.9. Bioinformatics Analysis

Quality control, read mapping, and counting were performed using PPLine pipeline [32]. Briefly, reads were analyzed using FastQC and then read trimming and adaptor removing were performed with trimmomatic [33]. The reads were mapped to the human genome (assembly hg38, Ensembl release 88) using STAR (1-pass mode) [34]. Approximately 87–92% reads were uniquely mapped. Since the samples had demonstrated different RNA integrity numbers (RIN), in order to compare gene expression levels, we took into account 3’-tail bias as described in [35] with some modifications. Without bias adjustment, one should expect false-positive overexpression of short transcripts (such as mRNAs encoding mitochondrial or ribosomal proteins) and under-expression of genes with long transcripts and encoded proteins (i.e., multi-domain transmembrane proteins interacting with extracellular matrix).

Read coverage across transcript length was analyzed using a modified version of geneBody_coverage.py script, a part of RSeQC package [36]. In the samples with lower RIN numbers and more degraded RNA rapid falling down of read coverage level after 750–1000 bp from 3’-tail was observed. The coverage in the region of 0–750 bp from 3’-tail was preserved for all the samples.

Then, transcripts were quantified using RSEM [37] and the most abundantly expressed alternative transcript for each gene was selected. The other transcripts were discarded. Then, the transcripts were truncated to the length 750 bp from 3’-end and new GTF file was generated. Finally, using this gene model, were quantified reads using featureCounts from the Subread package [38]. This procedure allowed us to significantly reduce the dependence of expression level fold change (FC) between higher and lower RINs on transcript length (e.g., impact of RIN differences). In order to completely eliminate this factor, we proceed the following way. All the genes were split into 10 bins depending on the average gene transcript length, and within each bin, we normalized read counts using TMM (trimmed mean of M-values) method from edgeR [39]. The similar procedure was performed to eliminate the dependency of expression level FC on ‘absolute’ gene expression level (in terms of read counts per million, CPM). The adjusted read counts were analyzed by edgeR using exact test and quasi-likelihood F-test [39]. Gene Ontology, KEGG, Reactome enrichment was performed using topGO and clusterProfiler Bioconductor packages for top-50, 100, 250, 500 and 1000 either up- or down-regulated genes [40]. KEGG pathways visualization was performed using a pathview package [41]. Raw FASTQ Illumina sequence data have been deposited at the Sequence Read Archive (SRA) under the accession number PRJNA494568. The dataset includes 12 records (from SAMN10172596 to SAMN10172607). For each sample, three replicates were sequenced.

### 2.10. Scratch Assay

HepaRG cells were grown on 6-well plates and treated with 10 μM DENSpm for 72 h. Then, the cell monolayer was scratched with a sterile p200 pipet tip, the debris was removed by changing the medium, and images of the cells were obtained immediately and 24 h later.

### 2.11. Measurement of ROS Production

HepaRG cells were grown on 24-well plates and treated with 10 μM DENSpm for 72 h in the absence or presence of 2.5 mM NAC or 100 μM trolox. Then, the medium was changed to the fresh medium supplemented with 10 μM 2′,7′-dichlorodihydrofluoresceine diacetate (DCFHDA), incubated for 30 min, washed with the fresh medium (10 × 0.5 mL), and fluorescence intensities were recorded on a Plate CHAMELEON V reader (Hidex, Turku, Finland) with excitation at 485 nm and emission at 535 nm.

### 2.12. Statistical Analysis

Statistical analysis was performed with XLStat software (Addinsoft, Paris, France). All the data are presented as means ± SD, *n* = 3–6. Significant differences were determined using a non-parametric Kruskal–Wallis test with Multiple pairwise comparisons using the Conover-Iman procedure [42]. A *p*-value < 0.05 was considered statistically significant if not stated otherwise.

## 3. Results

### 3.1. Unlike Huh7, HepG2, and SkHep1 Cells, HepaRG Cells Exhibit Elevated Expression Levels of Hepatocyte-Specific Genes

**Figure 1:**
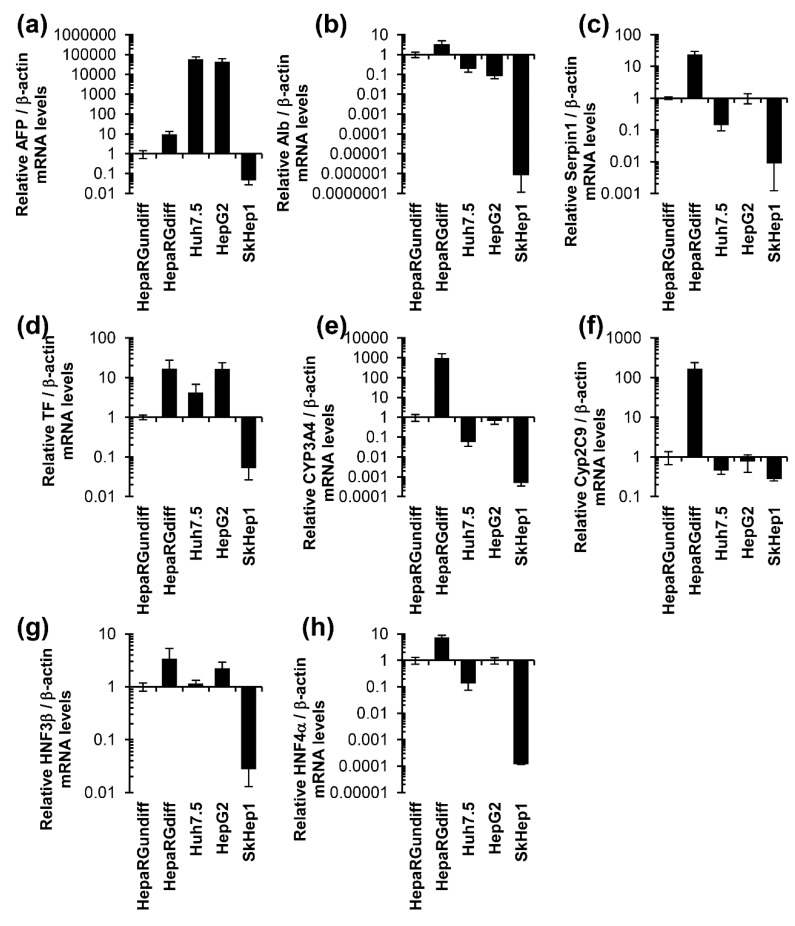
HepaRG cells compared to Huh7.5, HepG2 and SkHep1 cells exhibit highest expression of hepatocyte-specific genes. Expression of genes in noncancer undifferentiated (HepaRG^undiff^) and differentiated (HepaRG^diff^) HepaRG, hepatocarcinoma Huh7.5 and HepG2 and adenocarcinoma SkHep1 cells was assessed by RT-qPCR, and the results were normalized to the level of β-actin mRNA. These resulting ratios were normalized to the values obtained for undifferentiated HepaRG cells. The genes include α-fetoprotein (AFP) (**a**), albumin (Alb) (**b**), α-antitrypsin (Serpin 1) (**c**), transferrin (TF) (**d**) cytochromes P450 3A4 (CYP3A4) (**e**) and 2C9 (**f**), hepatocyte nuclear factors 3β (HNF3β) (**g**) and 4α (HNF4α) (**h**). Statistical analysis is presented in the Supplementary Table S3).

Expression of a number of hepatocyte specific genes was compared in the transformed hepatoma cell lines Huh7 and HepG2, the adenocarcinoma cell line SkHep1, and the non-transformed hepatic progenitor HepaRG cell line that can be differentiated into a mixture of hepatocytes and cholangiocytes. Differentiation of HepaRG cells was achieved by culturing the cells at confluency for two weeks in standard Williams E medium followed by two weeks in Williams E medium supplemented with DMSO. It has been reported that the differentiated HepaRG (HepaRG^diff^) cells exhibit elevated levels of hepatocyte-specific genes compared to hepatocarcinoma HepG2 cells (for example, [25,43]). To validate HepaRG as an appropriate cell line for this study, expression levels of a number of hepatocyte-specific genes were compared in the four cell lines, including albumin, cytochromes (CYP) 3A4 and 2C9, alpha-antitrypsin (AAT, Serpin1), transferrin (TF), hepatic nuclear factors (HNF) 3b and 4A as well as a marker of liver carcinoma—α-fetoprotein (aFP). In all cases the values were normalized to the levels of expression of the corresponding gene in undifferentiated HepaRG cells (HepaRG^undiff^). As shown in Figure 1, HepaRG^diff^ exhibit markedly elevated levels of almost all of the differentiation markers, compared to Huh7.5 and HepG2 cells as well as to HepaRG^undiff^. Particularly elevated differences were observed for CYP3A4 and CYP2C9 (Figure 1e,f). In contrast, expression levels of aFP were several orders of magnitude lower in HepaRG cells, compared to the transformed hepatic cell lines. Thus, HepaRG^diff^ cells expressed much higher levels of markers of mature hepatocytes than the hepatoma cell lines Huh7.5 or HepG2.

Transition from proliferation to a quiescent state and concomitant differentiation of HepaRG cells was also accompanied by a strong reduction in the levels of all polyamines. In fact, putrescine and spermidine levels became barely detectable in HepaRG^diff^ (Table 1). Only spermine remained detectable, but at over 20 fold reduced levels compared to HepaRG^undiff^. These changes were not due to loss of cell proliferation: HepaRG cells kept for two weeks at confluency in standard Williams medium, prior to DMSO addition, exhibited lower levels of spermine (32 ± 7 nmol/mg DNA) and spermidine (21.3 ± 6.7 nmol/mg DNA) than HepaRG^undiff^ but higher levels than those in HepaRG^diff^. The differentiation process also altered the relative expression levels of spermine and spermidine, relatively balance in hepatoma Huh7.5 and HepG2 cell lines, towards a strong prevalence of spermine in HepaRG^diff^. In contrast, SkHep1 cells, that are of endothelial origin, exhibited a lower level of spermine compared to spermidine (Table 1). HepaRG^diff^ exhibit not only higher expression levels of hepatocyte-specific genes but also a higher abundance of spermine over spermidine, but an overall much lower polyamine content compared to cancer-derived cell lines.

### 3.2. Diethylnorspermine Induces Dedifferentiation of HepaRG Cells and Mesenchymal-Like Phenotype

**Figure 2:**
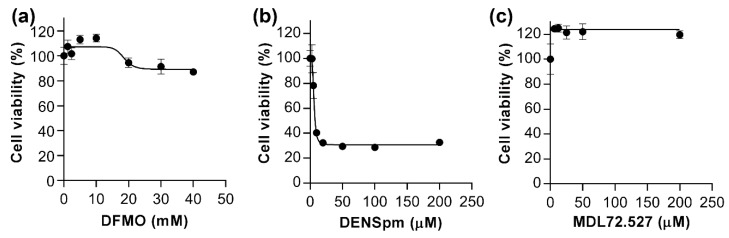
Toxicity of DFMO, DENSpm, and MDL72.527. Differentiated HepaRG cells were incubated with 1–40 mM DFMO (**a**), 3–200 μM DENSpm (**b**), or 6–200 μM MDL72.527 (**c**) for 72 h, and cell viability was assessed by standard MTT test. Results are presented as means ± S.D. from three experiments.

To investigate if alteration of polyamine metabolism can trigger EMT, three compounds were used that deregulate different stages of polyamine biosynthesis or degradation. DFMO, an ODC inhibitor, blocks synthesis of putrescine; DENSpm, a SSAT inducer, augments spermine and spermidine catabolism; and MDL72.527 down-regulates polyamine degradation due to the inhibition of both APAO and SMO [8]. First, cytotoxic effects of these compounds on HepaRG^diff^ cells were analyzed using an MTT test, where MTT is converted into the purple formazan product by oxidoreductases contained only in living cells [28]. DFMO was nontoxic to the cells up to 40 mM (Figure 2a). DENSpm caused a strong cytotoxic effect at micromolar concentrations in these cells (Figure 2b), which acquired a dedifferentiated phenotype (not shown). Surprisingly, ca. 30% of initial amount of cells were resistant to DENSpm and a further increase of DENSpm concentration from 10 μM to 200 μM did not enhance cell death in this resistant population. Finally, MDL72.527 caused an increase of MTT conversion into the formazan product at all concentrations (6–200 μM) (Figure 2b). However, since these cells were already at 100% confluency and could not grow further, this increase may reflect enhanced mitochondrial metabolic processes, uncoupling, or other metabolic changes rather than cell proliferation [44,45]. Therefore, for further studies 5 mM DFMO, 25 µM MDL72.527, and 10 µM DENSpm were chosen as working concentrations.

**Figure 3:**
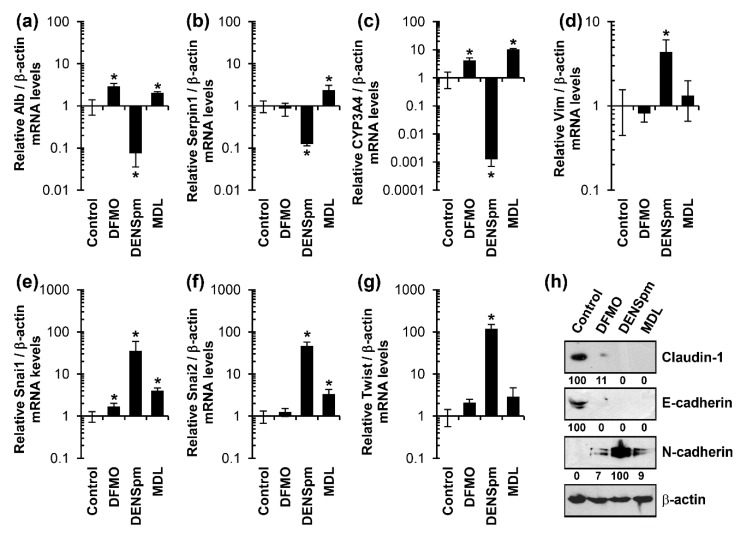
Diethyl-nor-spermine causes a decrease in expression of hepatocyte-specific genes and an increase in expression of mesenchymal cell-specific genes in hepatic epithelial HepaRG cells. Differentiated HepaRG cells were treated for 72 h with 5 mM DFMO, 10 μM DENSpm, or 25 μM MDL72.527 (denoted on the figure as MDL). (**a**–**g**) Quantification of levels of mRNAs for liver epithelial and mesenchymal cells were performed as described above. The results are presented as means ± S.D. from three independent experiments. * *p* < 0.05 vs. untreated cells (Control). (**h**) Expression of epithelial (claudin-1, E-cadherin) and mesenchymal (N-cadherin)—specific genes was quantified by western blotting, using β-actin as a house-keeping gene. The values represent protein/β-actin levels normalized to the control (%) with the exception of N-cadherin.

HepaRG^diff^ were treated with these compounds for 72 h, and EMT was monitored by quantifying levels of hepatocyte-specific genes (albumin, CYP3A4, and α-AT) or markers of mesenchymal cells (vimentin, Snai1, Snai2, and Twist) by RT-qPCR. In addition, expression of tight and adhesion junction proteins claudin 1, E-cadherin and ZO-1, and the mesenchymal cell marker N-cadherin were assessed by Western blotting. Treatment of HepaRG^diff^ with 5 mM DFMO or 25 µM MDL72.527 resulted in a decrease in expression of junction proteins (Figure 3h) but had little if any influence on expression of any other genes (Figure 3a–g). In contrast, 10 µM DENSpm induced a strong suppression of albumin, CYP3A4 and α-AT (Figure 3a–c). In addition, this compound also triggered expression of mesenchymal cell markers in a reproducible fashion (Figure 3d–h). Therefore, DENSpm appeared to induce an epithelial-mesenchymal transition.

*Bona fide* EMT is characterized by increased migratory capacity. Therefore, the ability of DENSpm to increase cell migration and evasion was tested. First, HepaRG^diff^ on 6-well plates were treated with DFMO, MDL72.527, or DENSpm. 24 h later a scratch was made in each well, and wound healing was monitored by microscopy. Neither untreated nor treated cells exhibited notable migration, with no difference between them (not shown). Absence of notable migration even after 48 h indicated the absence of a mesenchymal phenotype in these cells. Therefore, the loss of differentiation markers can be referred to as an EMT-like dedifferentiation.

### 3.3. Antioxidants Trolox and N-Acetylcysteine Do Not Affect Altered Expression of Epithelial or Mesenchymal Cell-Specific Genes

**Figure 4:**
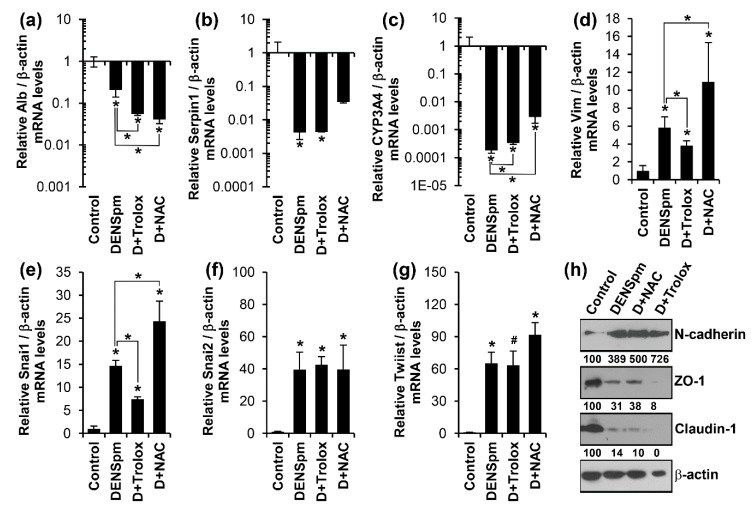
DENSpm-triggered deddifferentiation is not mediated by reactive oxygen species. Differentiated HepaRG cells were treated for 72 h with 10 μM DENSpm in the absence or presence of 100 μM trolox (D+Trolox) or 2.5 mM N-acetylcysteine (D+NAC). (**a**–**g**) Quantification of levels of mRNAs for liver epithelial and mesenchymal cells were performed as described above. The results are presented as means ± S.D. from three independent experiments. * *p* < 0.05 vs. untreated cells if not stated otherwise (Control), # *p* = 0.06 vs. the untreated cells. (**h**) Expression of epithelial (claudin-1, ZO-1) and mesenchymal (N-cadherin)—specific genes were quantified by western blotting, using β-actin as a house-keeping gene. The values represent protein/ β-actin levels normalized to the control (%).

In many cases EMT is driven by reactive oxygen species (ROS), as was clearly revealed for TGFβ1—the classical EMT inducer [46]. Since DENSpm-induced polyamine catabolism is accompanied by the production of hydrogen peroxide and N-acetyl-3-aminopropanal as stochiometric by-products, the next goal was to check if these latter compounds also play a significant role in drug-induced dedifferentiation of HepaRG^diff^. To test this hypothesis, two antioxidants—trolox and N-acetylcysteine (NAC) were used. Noteworthy, NAC not only neutralizes ROS but also toxic acrolein [47,48]. However, the addition of neither trolox nor NAC prevented the suppression of hepatocyte-specific genes in DENSpm-treated HepaRG^diff^ (Figure 4a–c). In addition, neither trolox nor NAC blocked the induction of genes typical for mesenchymal cells (Figure 4d–g). Of note, treatment of HepaRG cells with DENSpm did not result in an apparent elevation of ROS levels, measured with the widely used DCFHDA (Supplementary Figure S1). This latter observation may not reflect the absence of enhanced polyamine catabolism but merely be the result of either efficient scavenging of peroxide in peroxisomes where acetylpolyamine oxidase (APAO) is localized or by other factors. This is in line with observations of several other groups that also did not observe any increase in DCF fluorescence upon SSAT overexpression [49,50]. Thus, DENSpm-triggered EMT was not mediated by overproduction of reactive oxygen species or aldehydes.

### 3.4. DENSpm Triggers Dedifferentiation of HepaRG Cells through Exhaustion of Spermidine

**Figure 5:**
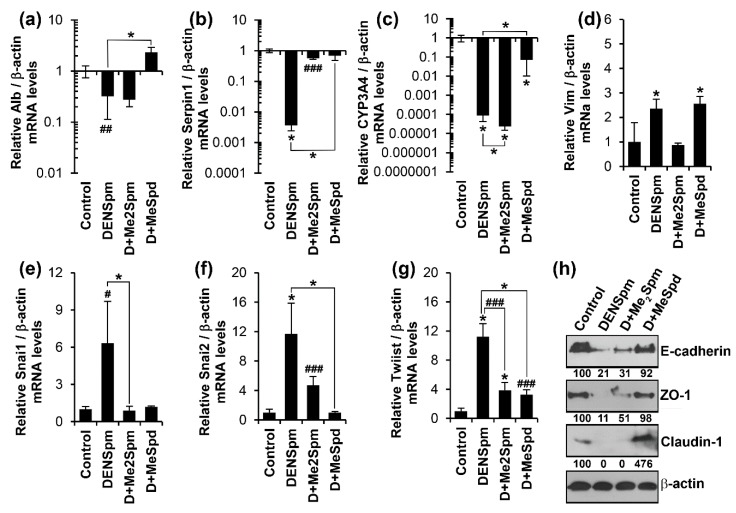
Metabolically-stable 3-methylspermidine and (S,S)-3,10-dimethylspermine prevent DENSpm-driven dedifferentiation of HepaRG cells. Differentiated HepaRG cells were treated for 72 h with 10 μM DENSpm in the absence or presence of 100 μM (R)-3-methylspermidine (D+MeSpd) or 100 μM (*R*,*R*)-1,12-dimethylspermine (D+Me2Spm). (**a**–**g**) Quantification of levels of mRNAs for liver epithelial and mesenchymal cells were performed as described above. The results are presented as means ± S.D. from three independent experiments. * *p* < 0.05, # *p* < 0.06, ## *p* < 0.08, ### *p* < 0.10 vs. untreated cells (Control) if not state otherwise. (**h**) Expression of claudin-1, E-cadherin and ZO-1 was quantified by western blotting, using β-actin as a house-keeping gene. The values represent protein/β-actin levels normalized to the control (%).

The next goal was to ask whether DENSpm-induced EMT could result from a decrease in polyamine content. To do this, the metabolically stable but functionally active methylated polyamines were used. It was previously shown that (*R*)-γ-methylspermidine (γ-MeSpd) can act as natural spermidine, whereas (*R*,*R*)-α,α′-dimethylspermine (α,α′-Me_2_Spm) can take over cellular functions of spermine [51,52], as exemplified by restoration of cell growth during depletion of natural polyamines. Addition of the 3-MeSpd to HepaRG^diff^ together with DENSpm for 72 h, prevented reduction of epithelial cell markers such as albumin, α-AT, or CYP3A4 (Figure 5a–c) as well as reduction of cell surface receptors E-cadherin, claudin 1 and ZO-1 (Figure 5h). 3-MeSpd also abolished induction of the EMT-associated transcription factors Snai1, Snai2, and Twist (Figure 5e–g). The only exception was expression of vimentin (panel D). DENSpm-induced changes were also prevented by Me_2_Spm (panels b, d, e, and g). However, for albumin, CYP3A4, Snai2, and cell surface receptors this mimetic of spermine exhibited much weaker or no effect (panels A, C, F, and H). The effect of DENSpm on albumin and Snai1 genes as well as of Me_2_Spm on α-AT, Twist, and Snai2 genes expression reached almost statistical significance (Figure 5). These findings suggest that polyamines control the differentiation status of hepatocytes, and that the functions of spermine and spermidine do not fully overlap in that respect.

### 3.5. Transcriptome Analysis

**Figure 6:**
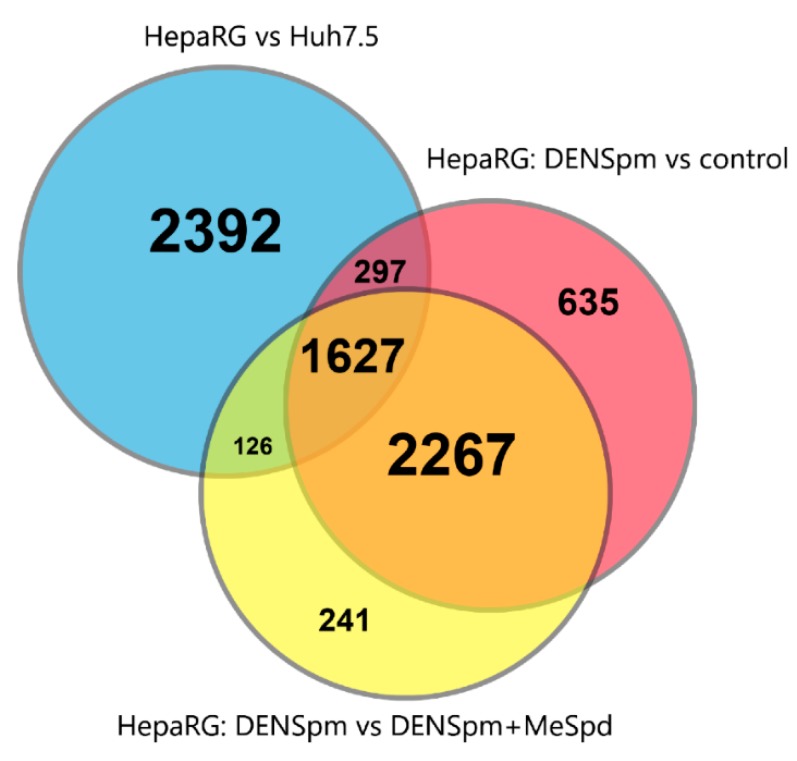
Venn diagram demonstrating the overlaps between the lists of differentially expressed genes for three comparisons.

**Figure 7:**
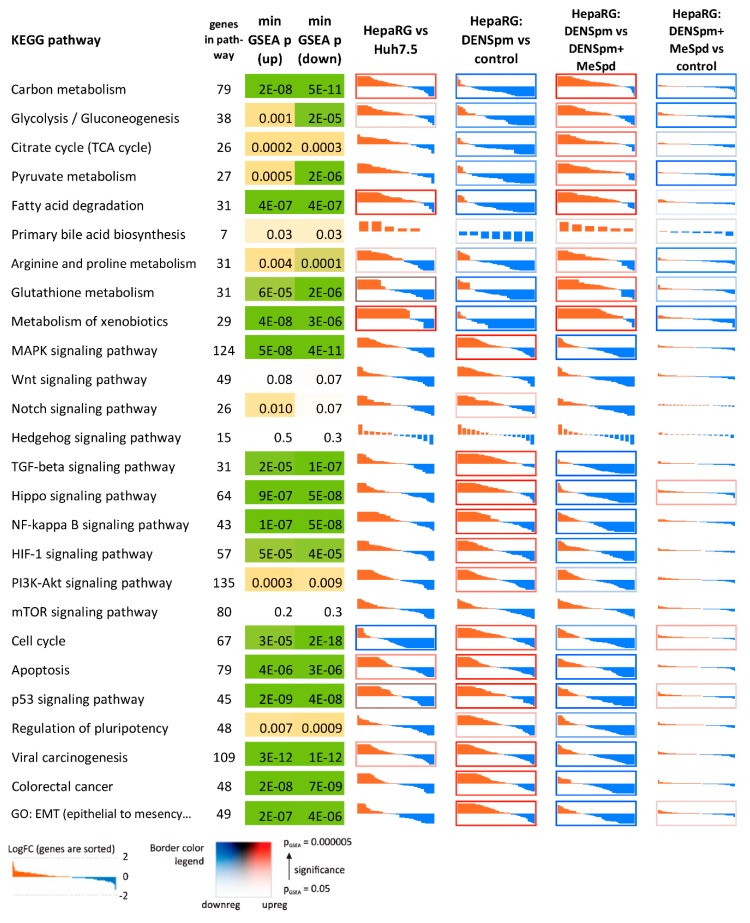
Differential expression profiles of genes involved in the key cell signaling pathways processes (KEGG pathways and two GO terms). Each cell represents log-transformed expression level fold changes (LogFC) of individual genes, sorted from overexpressed (red) to downregulated (blue). In each cell, vertical axis range is from LogFC = −2 (i.e., 4-fold downregulation; blue) to LogFC = +2 (i.e., 4-fold overexpression; red). Cell borders indicate the gene set enrichment test (GSEA) *p*-value: blue borders—enrichment with down-regulated genes, red borders—with overexpressed genes. ‘min. GSEA p (up/down)’—minimal GSEA *p*-value for up/down-regulated genes (across all the analyses).

**Figure 8:**
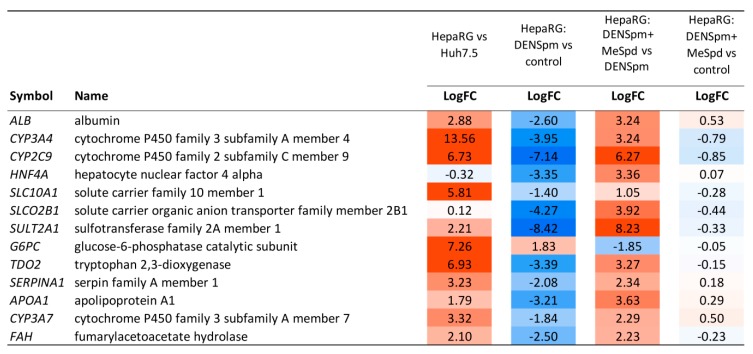
Expression of mature hepatocyte markers in four comparisons: (a) HepaRG vs. Huh7.5 cells (HepaRG vs. Huh7.5); (b) DENSpm-treated HepaRG vs. non-treated HepaRG cells (HepaRG: DENSpm vs. control); (c) DENSpm+MeSpd-treated HepaRG cells vs. DENSpm-treated HepaRG (HepaRG: DENSpm+MeSpd vs. DENSpm); (d) DENSpm+MeSpd-treated HepaRG cells vs. non-treated HepaRG cells (HepaRG: DENSpm+MeSpd vs. control). LogFC—binary logarithm of expression level fold change.

**Figure 9:**
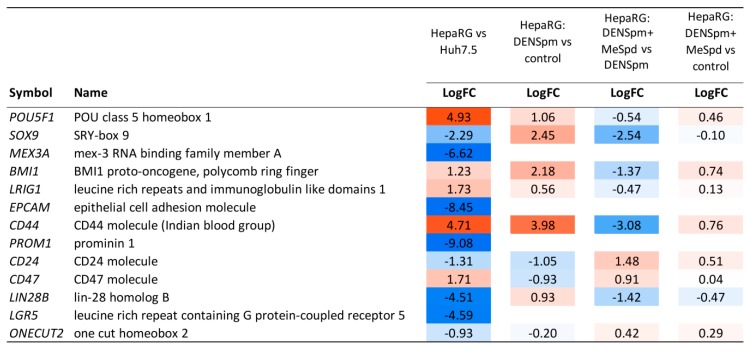
Expression of stem cell marker genes in four comparisons: (a) HepaRG versus Huh7.5 cells (HepaRG vs. Huh7.5); (b) DENSpm-treated HepaRG vs. non-treated HepaRG cells (HepaRG: DENSpm vs. control); (c) DENSpm+MeSpd-treated HepaRG cells vs. DENSpm-treated HepaRG (HepaRG: DENSpm+MeSpd vs. DENSpm); (d) DENSpm+MeSpd-treated HepaRG cells vs. non-treated HepaRG cells (HepaRG: DENSpm+MeSpd vs. control). LogFC—binary logarithm of expression level fold change.

**Figure 10:**
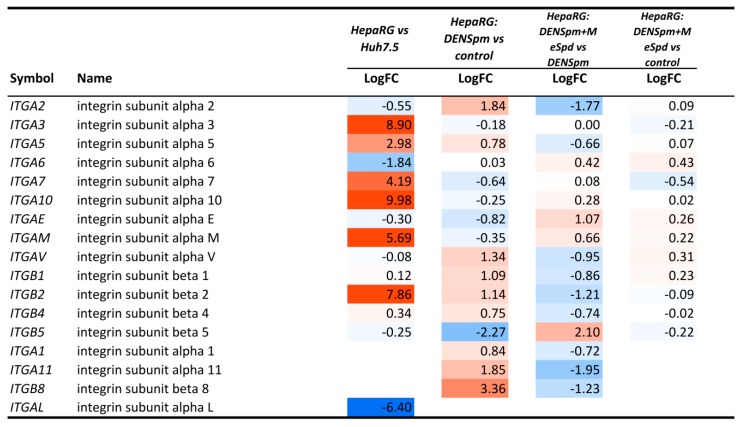
Expression of genes encoding integrins in four comparisons: (a) HepaRG vs. Huh7.5 cells (HepaRG vs. Huh7.5); (b) DENSpm-treated HepaRG vs. non-treated HepaRG cells (HepaRG: DENSpm vs. control); (c) DENSpm+MeSpd-treated HepaRG cells vs. DENSpm-treated HepaRG (HepaRG: DENSpm+MeSpd vs. DENSpm); (d) DENSpm+MeSpd-treated HepaRG cells vs. non-treated HepaRG cells (HepaRG: DENSpm+MeSpd vs. control). LogFC—binary logarithm of expression level fold change.

To further delineate the roles of polyamines in HepaRG differentiation, a comparative transcriptome approach based on next-generation RNA sequencing (RNA-seq) followed by bioinformatics analysis was undertaken. The results are presented on Figure 6, Figure 7, Figure 8, Figure 9 and Figure 10 and Supplementary Tables S4–S11. Comparison of HepaRG^diff^ with Huh7.5 cells revealed dramatic transcriptional differences: 4442 differentially expressed genes that passed the following thresholds: fold change >2 or <0.5, read counts per million (CPM) > 4, false discovery rate (FDR) < 0.01 (Venn diagram on Figure 6). As can be seen on Figure 7, HepaRG^diff^ exhibited elevated expression of genes of a variety of metabolic pathways including carbon metabolism, TCA cycle, glycolysis and gluconeogenesis, pyruvate metabolism and fatty acid degradation (Figure 7). In addition, they demonstrated significantly enhanced transcription of genes involved in primary bile acid biosynthesis (i.e., markers of cholangiocytes) and of those involved in metabolism of xenobiotics. A majority of the latter are cytochromes P450, highly expressed in mature hepatocytes. Figure 8 illustrates the differential expression of hepatocyte specific genes including those analyzed by standard RT-qPCR (Figure 1). Again, HepaRG^diff^ display significantly higher levels of their expression, compared to Huh7.5 cells (Figure 8).

Treatment of HepaRG^diff^ cells with DENSpm induced a huge transcriptome shift, the extent of which is comparable to the differences between HepaRG^diff^ and Huh7.5 cells described above. The expression of 4826 genes was affected (fold change > 2 or <0.5, read counts per million (CPM) > 4, false discovery rate (FDR) < 0.01, Figure 6). Bioinformatic analysis showed that activation of polyamine catabolism led to down-regulation of genes of various metabolic pathways (TCA cycle, glycolysis, fatty acid degradation, bile acid biosynthesis, amino acid metabolism) but upregulated expression of genes from various signaling pathways including EMT, TGF-beta, MAPK and other ones (Figure 7). In lines with our results shown on Figure 3, DENSpm caused a strong down-regulation of hepatocyte-specific genes (see Figure 8, and “metabolism of xenobiotics” pathway on Figure 7). In addition, DENSpm-treated cells exhibited elevated levels of expression of several key stem cell markers including POU5F1, Sox9, CD44, BMI1, and Lin28B, although other transcripts that are found in liver stem and cancer stem cells remained absent (PROM1, CD133) (Figure 9). Almost all these changes were blocked by 3-MeSpd: a comparison of HepaRG cells treated with a combination of DENSpm and MeSpd with control HepaRG cells showed only minor differences in gene expression patterns (Figure 7). Only 34 genes have passed the differential expression thresholds described above. Noteworthy our RT-qPCR and transcriptome analysis are consistent. Thus, next-generation sequencing confirmed that activation of polyamine catabolism in hepatocyte-like HepaRG cells triggers an EMT-like dedifferentiation. 

The conclusion that the process is not a *bona fide* EMT was based on absence of evidence that DENSpm enhanced migration capacity of the HepaRG cells. As cell motility is tightly linked to expression of integrins [53], an analysis of expression of various α- and β- isoforms in the transcriptomic dataset. The results are presented on Figure 10 and Supplementary Table S11. It can be seen that the HepaRG^diff^ cells exhibit significantly higher levels of integrins α3, α5, α7, α10, and β2 that Huh7.5 cells. DENSpm-triggered dedifferentiation led to additional increase of α5, β1, β2, and β4 integrins. At the same time, in HepaRG cells several integrins were not detected: α1, α4, α9 and β3. Their absence could explain the non-migrating phenotype of the HepaRG^diff^ cells.

## 4. Discussion

HepaRG cells, in contrast to the widely used Huh7 and HepG2 hepatoma cell lines, are non-transformed cells, and their metabolism closely resembles that of normal primary hepatocytes, characterized by a strong secretory activity (albumin) and secretion of vLDL (apoB ELISA) [54]. Our experiments confirm that HepaRG exhibit profoundly elevated levels of expression of cytochromes P450 3A4 and 2C9, albumin and α-antitrypsin compared to cancer cell lines. In addition, HepaRG^diff^ demonstrated markedly lower polyamine levels compared to hepatoma cell lines, known to contain elevated pools of spermine and spermidine. It also correlates with the fact that these differentiated cells do not proliferate, being confluent, whereas polyamine levels usually correlate with proliferation rates.

Biogenic polyamines have already been implicated in differentiation of several types of mammalian cells. However, they can play either positive or negative roles depending on the cell type. For example, polyamines have been implicated in chondrocyte differentiation (reviewed in [55]). Exogenous polyamines that triggered induction of SSAT and suppression of ODC induction suppress differentiation of human bone marrow-derived mesenchymal stem cells (hBMSCs) into adipocytes but enhance differentiation into osteoblasts [56]. Similar results were also reported in the case of ODC down-regulation by RNAi or inhibition using DFMO [57]. Overexpression of SSAT in mice altered the hematological phenotype by inducing proliferation of myeloid cells [58] as well as impaired osteoblastogenesis [59]. Among other cell types, whose differentiation is dependent on polyamines are myoblasts [60], germ cells [61], and keratinocytes [62]. Our data expand this list to liver epithelial cells. In addition, we have shown that dedifferentiation of HepaRG cells can be caused by a decrease in spermidine level, whereas spermine was found dispensable for this process. Both spermine and spermidine are readily converted into each other thus making it difficult to discriminate between their functions. In our work we used two compounds, namely γ-MeSpd and α,α′-Me_2_Spm, that can substitute the biological functions of biogenic polyamines but that cannot be converted into other polyamine analogues [51,52]. Hyvonen et al. have used these compounds to show that spermidine but not spermine is indispensable for differentiation of 3T3-L1 fibroblasts into adipocytes [63,64]. These data correlate with similar findings of Ishii et al. who used an alternative approach in changing Spm/Spd ratio that consisted in inhibiting either spermine or spermidine synthases [65]. Finally, Alhonen et al. reported that liver regeneration in SSAT-transgenic rats is more strongly associated with levels of spermidine than of spermine [66]. In our case, on one hand, the differentiated HepaRG cells exhibited lower total levels of spermine and spermidine than the undifferentiated, and DENSpm-triggered dedifferentiation lead to further decrease in their content. On the other hand, in HepaRG^undiff^ as well as in highly metastatic SkHep1 cells the ratio between spermidine and spermine was ≥1, and DENSpm also significantly shifted ratio between spermine and spermidine with the latter to become the predominant one.

It is tempting to speculate that our results could be linked to liver regeneration. To date several possible mechanisms of liver regeneration were proposed. First, it can occur via proliferation of hepatocytes. Earlier this year Oh et al. reported that liver regeneration requires EMT in hepatocytes, as evidenced by enhanced expression of αSMA, collagen1A1, MMP9, and ZEB1 as well as by TGFβ signaling [67]. Our study shows that DENSpm up-regulated expression of the ACTA2 gene (encodes smooth muscle actin), ZEB2, TGBβ3 and several MMP isoforms (Table S4). Second, regeneration can be achieved by formation of hepatocytes from hepatic progenitor cells of biliary origin, as was shown in vivo in the context of massive senescence of initial mass of hepatocytes [68]. It has been proposed that such liver progenitor cells can give rise to both epithelial and mesenchymal cells (such as hepatic stellate cells, or HSCs) through formation of two subpopulations, each of them differentiating into either parenchymal or non-parenchymal cells [69]. HepaRG cells represent a unique bipotent hepatic progenitor line that can transdifferentiate into a mixed culture of epithelial cells; i.e., hepatocytes and cholangiocytes [23,24,25]. Therefore, HepaRG cells may represent such a segregated population, which means that the dedifferentiation shown here could be linked to liver development or/and regeneration processes. Recently hepatic stem-like cells were identified as a source of parenchymal cells even in the absence of tissue damage, an additional argument in favor of our assumption [70]. Previous data of the Alhonen laboratory demonstrated that enhanced polyamine catabolism in rats due to SSAT overexpression blocked liver regeneration, implying role of polyamines in hepatocyte proliferation [66]. Our data may provide insight of role of spermidine in other mechanism of regeneration of this organ. 

Currently, the mode of action of spermidine in cell differentiation remains unknown. Spermidine is a precursor of a unique type of post-translational protein modification—hypusination that is a feature of eIF5α. So, altered levels of spermidine may affect the protein translation machinery and in particular levels of proline-rich proteins as one of eIF5α functions in translation elongation of polyproline stretches [71] which are abundant and critical for functioning of many signal transducing proteins [72]. Another feature of spermidine is its conversion into N^8^-acetylspermidine that is involved in regulation of histone acetylases [73].

Our initial goal was to find a role of biogenic polyamines in epithelial-mesenchymal transition (EMT). However, our results show that modulation of polyamine metabolism does not result into a *bona fide* EMT but rather an EMT-like dedifferentiation, judged by the absence of motility of DENSpm-treated cells. It is accepted that cell migration capacity is tightly associated with the expression of integrin family members [53]. Integrins form heterodimes composed of α- and β-subunits. Transcriptomic analysis revealed that the HepaRG^diff^ exhibit elevated expression of several α- and β- integrins compared to Huh7.5 cells. At the same time, several other integrins such as α1, α4, α9 and β3 were not detected. However, the literature on the influence of polyamines on cell motility and on intergrin expression remains vague. On one hand, inhibition of polyamine biosynthesis with DFMO was reported to suppress migration of intestinal epithelial cells (reviewed in [74]). On the other, activation of polyamine catabolism through SSAT overexpression was shown to up-regulate cell motility [75]. The latter was mediated by direct interaction of SSAT with α9 subunit of the α9β1 integrin. So, it is tempting to speculate that the absence of migration of DENSpm-treated HepaRG cells continue, despite the induction of SSAT, which could be due to absence of integrin α9.

These findings warrant to study in more detail the contribution of polyamines to fibrosis in the liver, but also other organs such as the lungs, kidneys, and others. Furthermore, modulation of differentiation by polyamines might impact carcinogenesis during chronic liver disease. Indeed, altered differentiation of stem cell-like liver progenitor cells is known to contribute to the development of hepatocellular carcinoma (reviewed in [76]). Cancer stem cells are considered as tumor-initiating cells [77]. They are identified by the expression of a variety of stem cell markers including EpCAM, CD133 (PROM1), CD44, SOX2 and SOX9, BMI1, LIN28B, Oct4 (POU5F1) etc. Liver CSCs are represented by CD133^+^/EpCAM^+^ cells [78]. In our study we showed that the differentiated HepaRG cells exhibit lower levels of expression of a majority of such markers, compared to hepatoma Huh7.5 cells (Figure 9) that contain a population of cancer stem-like cells [76,77]. Activation of polyamine catabolism enhanced expression of several such markers including POU5F1, SOX9, CD44, BMI1, and LIN28B (Figure 9). However, several key markers including EpCAM and CD133 (PROM1) were not expressed. Thus, DENSpm-induced dedifferentiation does not give rise to liver cancer stem cells. This is in lines with the data from DENSpm clinical trials in patients with liver, breast, or lung cancer during which patients displayed occurrence of new tumors neither in liver nor other organs [10,11,12]. However, it could be speculated that activated polyamine catabolism may contribute to tumorigenesis, if accompanied by other cancerogenic stimuli. Hepatitis C virus (HCV) accounts together with hepatitis B virus (HBV) for a majority of cases of liver cancer [79]. Recently, we have shown that transient expression of HCV proteins leads to SSAT induction, whereas a long lasting “chronic” replication of the virus is accompanied by induction of SMO, downregulation of ODC and SSAT and, as a result, by a decrease in spermine and spermidine pools [80]. Thus, the pro-carcinogenic potential of HCV proteins, the profibrotic activity of viral infection and dysregulated polyamine levels may synergistically contribute to/augment occurrence of hepatocellular carcinoma.

Finally, our data clearly demonstrate that cell (de)differentiation is tightly linked to various metabolic pathways, and their deregulation may be a trigger of defifferentiation. Specifically, it was shown that polyamines, and spermidine in particular, are important factors of differentiation of hepatocytes, and that activation of polyamine catabolism causes an EMT-like dedifferentiation. In lines with multiple reports from different teams, such dedifferentiation/EMT is accompanied by down-regulation of oxidative phosphorylation system, fatty acids, and bile catabolic systems, peroxisomal processes etc. (as can be exemplified by [81]). At the same time, in our model we did not register any signs of other EMT-accompanying metabolic changes such as enhanced glycolysis and glutaminolysis [82]. So, these data illustrate that EMT and EMT-like dedifferentiation of non-tumor cells are associated with significant metabolic adaptation, however there are notable differences between these two events in the exact implicated pathways. Investigation of these differences in other models merits further studies.

## 5. Conclusions

Taken together, we show here that activation of polyamine catabolism in non-cancerous epithelial liver HepaRG cells caused a dedifferentiation and a mesenchymal-like phenotype. Importantly, this process was due not to increased ROS production but resulted from the exhaustion of the spermidine level. These data provide additional pieces of evidence that altered metabolism of biogenic polyamines can contribute to liver pathology.

## Figures and Tables

**Table 1:** Polyamine levels in cell lines.

Cell Line	Put (nmol/mg DNA)	Spd (nmol/mg DNA)	Spm (nmol/mg DNA)	N1-AcSpd (nmol/mg DNA)
Huh7.5 ^1^	N.D. ^2^	17.8 ± 5.9	63.2 ± 15.0	N.D. ^2^
HepG2 ^1^	N.D. ^2^	20.7 ± 5.8	54.2 ± 16.5	8.5 ± 1.5
HepaRG^undiff 1^	10.4 ± 1.0	54.8 ± 6.3	53.9 ± 3.7	N.D. ^2^
HepaRG^diff 3^	N.D. ^2^	N.D. ^2^	2.6 ± 0.3	N.D. ^2^
SkHep1 ^1^	9.9 ± 4.3	25.0 ± 7.7	16.5 ± 3.4	N.D. ^2^

^1^ At subconfluent state. ^2^ N.D.—Not detected. ^3^ at confluent state after differentiation according the standard protocol.

## Supplementary Materials

The following are available online at http://www.mdpi.com/2073-4409/7/12/275/s1. Table S1: Primers used for quantification of gene transcription levels by real-time RT-PCR; Table S2: Polyamine levels in the differentiated HepaRG cells treated with DFMO, DENSpm or MDL72.527 for 72 h; Table S3: Statistical significance between groups, analyzed by Kruskal-Wallis method with Conover-Iman post-hoc approach; Table S4: Column annotations; Table S5: All analyses—Summary expression info.xlsx, joint lite, LogFC table; Table S6: HepaRG vs Huh7.5; Table S7: HepaRG—DENSpm vs. control; Table S8: HepaRG—DENSpm vs. DENSpm+MeSpd; Table S9: DENSpm+MeSpd vs. control HepaRG; Table S10: DENSpm+MeSpd vs. control HepaRG; Table S11: Integrins in all samples.

## References

[1] Bray F., Ferlay J., Soerjomataram I., Siegel R.L., Torre L.S., Jemal A. (2018). Global cancer statistics 2018: GLOBOCAN estimates of incidence and mortality worldwide for 36 cancers in 185 countries. CA Cancer J. Clin..

[2] Society A.C. (2018). Cancer Facts & Figures 2018.

[3] Morrot A., da Fonseca L.M., Salustiano E.J., Gentile L.B., Conde L., Filardy A.A., Franklim T.N., da Costa K.M., Freire-de-Lima C.G., Freire-de-Lima L. (2018). Metabolic symbiosis and immunomodulation: How tumor cell-derived lactate may disturb innate and adaptive immune responses. Front. Oncol..

[4] Pegg A.E., Casero R.A. (2011). Current status of the polyamine research field. Methods Mol. Biol..

[5] Janne J., Poso H., Raina A. (1978). Polyamines in rapid growth and cancer. Biochim. Biophys. Acta.

[6] Pegg A.E. (2009). Mammalian polyamine metabolism and function. IUBMB Life.

[7] Smirnova O.A., Bartosch B., Zakirova N.F., Kochetkov S.N., Ivanov A.V. (2018). Polyamine metabolism and oxidative protein folding in the ER as ROS-producing systems neglected in virology. Int. J. Mol. Sci..

[8] Casero R.A., Murray Stewart T., Pegg A.E. (2018). Polyamine metabolism and cancer: Treatments, challenges and opportunities. Nat. Rev. Cancer.

[9] Casero R.A., Pegg A.E. (2009). Polyamine catabolism and disease. Biochem. J..

[10] Goyal L., Supko J.G., Berlin J., Blaszkowsky L.S., Carpenter A., Heuman D.M., Hilderbrand S.L., Stuart K.E., Cotler S., Senzer N.N. (2013). Phase 1 study of n(1),n(11)diethylnorspermine (DENSPM) in patients with advanced hepatocellular carcinoma. Cancer Chemother. Pharmacol..

[11] Hahm H.A., Ettinger D.S., Bowling K., Hoker B., Chen T.L., Zabelina Y., Casero R.A. (2002). Phase I study of n(1),n(11)-diethylnorspermine in patients with non-small cell lung cancer. Clin. Cancer Res..

[12] Wolff A.C., Armstrong D.K., Fetting J.H., Carducci M.K., Riley C.D., Bender J.F., Casero R.A., Davidson N.E. (2003). A phase ii study of the polyamine analog n(1),n(11)-diethylnorspermine (DENSPM) daily for five days every 21 days in patients with previously treated metastatic breast cancer. Clin. Cancer Res..

[13] Creaven P.J., Perez R., Pendyala L., Meropol N.J., Loewen G., Levine E., Berghorn E., Raghavan D. (1997). Unusual central nervous system toxicity in a phase I study of n(1),n(11)-diethylnorspermine in patients with advanced malignancy. Investig. New Drugs.

[14] Saulnier Sholler G.L., Gerner E.W., Bergendahl G., MacArthur R.B., VanderWerff A., Ashikaga T., Bond J.P., Ferguson W., Roberts W., Wada R.K. (2015). A phase I trial of DFMO targeting polyamine addiction in patients with relapsed/refractory neuroblastoma. PLoS ONE.

[15] Samal K., Zhao P., Kendzicky A., Yco L.P., McClung H., Gerner E., Burns M., Bachmann A.S., Sholler G. (2013). Amxt-1501, a novel polyamine transport inhibitor, synergizes with dfmo in inhibiting neuroblastoma cell proliferation by targeting both ornithine decarboxylase and polyamine transport. Int. J. Cancer.

[16] Patel A.R., Li J., Bass B.L., Wang J.Y. (1998). Expression of the transforming growth factor-beta gene during growth inhibition following polyamine depletion. Am. J. Physiol..

[17] Rao J.N., Li L., Bass B.L., Wang J.Y. (2000). Expression of the TGF-beta receptor gene and sensitivity to growth inhibition following polyamine depletion. Am. J. Physiol. Cell Physiol..

[18] Liu L., Santora R., Rao J.N., Guo X., Zou T., Zhang H.M., Turner D.J., Wang J.Y. (2003). Activation of TGF-beta-SMAD signaling pathway following polyamine depletion in intestinal epithelial cells. Am. J. Physiol..

[19] Guo X., Rao J.N., Liu L., Zou T., Keledjian K.M., Boneva D., Marasa B.S., Wang J.Y. (2005). Polyamines are necessary for synthesis and stability of occludin protein in intestinal epithelial cells. Am. J. Physiol..

[20] Prunotto M., Compagnone A., Bruschi M., Candiano G., Colombatto S., Bandino A., Petretto A., Moll S., Bochaton-Piallat M.L., Gabbiani G. (2010). Endocellular polyamine availability modulates epithelial-to-mesenchymal transition and unfolded protein response in MDCK cells. Lab. Investig..

[21] Compagnone A., Bandino A., Meli F., Bravoco V., Cravanzola C., Parola M., Colombatto S. (2012). Polyamines modulate epithelial-to-mesenchymal transition. Amino Acids.

[22] Kalluri R., Weinberg R.A. (2009). The basics of epithelial-mesenchymal transition. J. Clin. Investig..

[23] Gripon P., Rumin S., Urban S., Le Seyec J., Glaise D., Cannie I., Guyomard C., Lucas J., Trepo C., Guguen-Guillouzo C. (2002). Infection of a human hepatoma cell line by hepatitis b virus. Proc. Natl. Acad. Sci. USA.

[24] Higuchi Y., Kawai K., Yamazaki H., Nakamura M., Bree F., Guguen-Guillouzo C., Suemizu H. (2014). The human hepatic cell line HEPARG as a possible cell source for the generation of humanized liver TK-NOG mice. Xenobiotica.

[25] Guillouzo A., Corlu A., Aninat C., Glaise D., Morel F., Guguen-Guillouzo C. (2007). The human hepatoma heparg cells: A highly differentiated model for studies of liver metabolism and toxicity of xenobiotics. Chem. Biol. Interact..

[26] Khomutov M.A., Keinanen T.A., Hyvonen M.T., Weisell J., Vepsalainen J., Alhonen L., Kochetkov S.N., Khomutov A.R. (2015). Enantioselective synthesis of (r)- and (s)-3-methylspermidines. Russ. J. Bioorg. Chem..

[27] Grigorenko N.A., Khomutov A.R., Keinanen T.A., Jarvinen A., Alhonen L., Janne J., Vepsalainen J. (2007). Synthesis of novel optical isomers of alpha-methylpolyamines. Tetrahedron.

[28] Rehse K., Puchert E., Leissring S. (1990). Antiaggregatory and anticoagulant effects of oligoamines. 12. Alkyl- and arylalkyl- derivatives of putrescine, spermidine and spermine. Arch. Pharm..

[29] Hyvonen T., Keinanen T.A., Khomutov A.R., Khomutov R.M., Eloranta T.O. (1992). Monitoring of the uptake and metabolism of aminooxy analogues of polyamines in cultured cells by high-performance liquid chromatography. J. Chromatogr..

[30] Ivanov A.V., Smirnova O.A., Petrushanko I.Y., Ivanova O.N., Karpenko I.L., Alekseeva E., Sominskaya I., Makarov A.A., Bartosch B., Kochetkov S.N. (2015). Hcv core protein uses multiple mechanisms to induce oxidative stress in human hepatoma huh7 cells. Viruses.

[31] Ivanov A.V., Smirnova O.A., Ivanova O.N., Masalova O.V., Kochetkov S.N., Isaguliants M.G. (2011). Hepatitis c virus proteins activate nrf2/are pathway by distinct ros-dependent and independent mechanisms in huh7 cells. PLoS ONE.

[32] Krasnov G.S., Dmitriev A.A., Kudryavtseva A.V., Shargunov A.V., Karpov D.S., Uroshlev L.A., Melnikova N.V., Blinov V.M., Poverennaya E.V., Archakov A.I. (2015). Ppline: An automated pipeline for snp, sap, and splice variant detection in the context of proteogenomics. J. Proteome Res..

[33] Bolger A.M., Lohse M., Usadel B. (2014). Trimmomatic: A flexible trimmer for illumina sequence data. Bioinformatics.

[34] Dobin A., Davis C.A., Schlesinger F., Drenkow J., Zaleski C., Jha S., Batut P., Chaisson M., Gingeras T.R. (2013). Star: Ultrafast universal RNA-seq aligner. Bioinformatics.

[35] Sigurgeirsson B., Emanuelsson O., Lundeberg J. (2014). Sequencing degraded RNA addressed by 3’ tag counting. PLoS ONE.

[36] Wang L., Wang S., Li W. (2012). RSEQC: Quality control of RNA-seq experiments. Bioinformatics.

[37] Li B., Dewey C.N. (2011). RSEM: Accurate transcript quantification from RNA-seq data with or without a reference genome. BMC Bioinform..

[38] Liao Y., Smyth G.K., Shi W. (2014). Featurecounts: An efficient general purpose program for assigning sequence reads to genomic features. Bioinformatics.

[39] Robinson M.D., McCarthy D.J., Smyth G.K. (2010). Edger: A bioconductor package for differential expression analysis of digital gene expression data. Bioinformatics.

[40] Yu G., Wang L.G., Han Y., He Q.Y. (2012). Clusterprofiler: An r package for comparing biological themes among gene clusters. OMICS.

[41] Luo W., Brouwer C. (2013). Pathview: An r/bioconductor package for pathway-based data integration and visualization. Bioinformatics.

[42] Conover W.J., Iman R.L. (1979). On Multiple-Comparisons Procedures.

[43] Kanebratt K.P., Andersson T.B. (2008). Evaluation of HEPARG cells as an in vitro model for human drug metabolism studies. Drug Metab. Dispos..

[44] Sylvester P.W. (2011). Optimization of the tetrazolium dye (MTT) colorimetric assay for cellular growth and viability. Methods Mol. Biol..

[45] Sliwka L., Wiktorska K., Suchocki P., Milczarek M., Mielczarek S., Lubelska K., Cierpial T., Lyzwa P., Kielbasinski P., Jaromin A. (2016). The comparison of MTT and CVS assays for the assessment of anticancer agent interactions. PLoS ONE.

[46] Liu R.M., Desai L.P. (2015). Reciprocal regulation of TGF-beta and reactive oxygen species: A perverse cycle for fibrosis. Redox Biol..

[47] Yoshida M., Tomitori H., Machi Y., Hagihara M., Higashi K., Goda H., Ohya T., Niitsu M., Kashiwagi K., Igarashi K. (2009). Acrolein toxicity: Comparison with reactive oxygen species. Biochem. Biophys. Res. Commun..

[48] Saiki R., Nishimura K., Ishii I., Omura T., Okuyama S., Kashiwagi K., Igarashi K. (2009). Intense correlation between brain infarction and protein-conjugated acrolein. Stroke.

[49] Ou Y., Wang S.J., Li D., Chu B., Gu W. (2016). Activation of sat1 engages polyamine metabolism with p53-mediated ferroptotic responses. Proc. Natl. Acad. Sci. USA.

[50] Lee D.Y., Chang G.D. (2014). Methylglyoxal in cells elicits a negative feedback loop entailing transglutaminase 2 and glyoxalase 1. Redox Biol..

[51] Hyvonen M.T., Khomutov M., Petit M., Weisell J., Kochetkov S.N., Alhonen L., Vepsalainen J., Khomutov A.R., Keinanen T.A. (2015). Enantiomers of 3-methylspermidine selectively modulate deoxyhypusine synthesis and reveal important determinants for spermidine transport. ACS Chem. Biol..

[52] Hyvonen M.T., Keinanen T.A., Cerrada-Gimenez M., Sinervirta R., Grigorenko N., Khomutov A.R., Vepsalainen J., Alhonen L., Janne J. (2007). Role of hypusinated eukaryotic translation initiation factor 5a in polyamine depletion-induced cytostasis. J. Biol. Chem..

[53] Wehrle-Haller B. (2000–2013). The role of integrins in cell migration. Madame Curie Bioscience Database.

[54] Cerec V., Glaise D., Garnier D., Morosan S., Turlin B., Drenou B., Gripon P., Kremsdorf D., Guguen-Guillouzo C., Corlu A. (2007). Transdifferentiation of hepatocyte-like cells from the human hepatoma HEPARG cell line through bipotent progenitor. Hepatology.

[55] Borzi R.M., Guidotti S., Minguzzi M., Facchini A., Platano D., Trisolino G., Filardo G., Cetrullo S., D’Adamo S., Stefanelli C. (2014). Polyamine delivery as a tool to modulate stem cell differentiation in skeletal tissue engineering. Amino Acids.

[56] Lee M.J., Chen Y., Huang Y.P., Hsu Y.C., Chiang L.H., Chen T.Y., Wang G.J. (2013). Exogenous polyamines promote osteogenic differentiation by reciprocally regulating osteogenic and adipogenic gene expression. J. Cell. Biochem..

[57] Tsai Y.H., Lin K.L., Huang Y.P., Hsu Y.C., Chen C.H., Chen Y., Sie M.H., Wang G.J., Lee M.J. (2015). Suppression of ornithine decarboxylase promotes osteogenic differentiation of human bone marrow-derived mesenchymal stem cells. FEBS Lett..

[58] Pirnes-Karhu S., Mantymaa P., Sironen R., Makinen P.I., Wojciechowski S., Juutinen S., Koistinaho J., Horkko S., Jantunen E., Alhonen L. (2014). Enhanced polyamine catabolism disturbs hematopoietic lineage commitment and leads to a myeloproliferative disease in mice overexpressing spermidine/spermine n(1)-acetyltransferase. Amino Acids.

[59] Pirnes-Karhu S., Maatta J., Finnila M., Alhonen L., Uimari A. (2015). Overexpression of spermidine/spermine n1-acetyltransferase impairs osteoblastogenesis and alters mouse bone phenotype. Transgen. Res..

[60] Cervelli M., Fratini E., Amendola R., Bianchi M., Signori E., Ferraro E., Lisi A., Federico R., Marcocci L., Mariottini P. (2009). Increased spermine oxidase (SMO) activity as a novel differentiation marker of myogenic c2c12 cells. Int. J. Biochem. Cell Biol..

[61] Quemener V., Blanchard Y., Lescoat D., Havouis R., Moulinoux J.P. (1992). Depletion in nuclear spermine during human spermatogenesis, a natural process of cell differentiation. Am. J. Physiol..

[62] Pietila M., Pirinen E., Keskitalo S., Juutinen S., Pasonen-Seppanen S., Keinanen T., Alhonen L., Janne J. (2005). Disturbed keratinocyte differentiation in transgenic mice and organotypic keratinocyte cultures as a result of spermidine/spermine n-acetyltransferase overexpression. J. Investig. Dermatol..

[63] Vuohelainen S., Pirinen E., Cerrada-Gimenez M., Keinanen T.A., Uimari A., Pietila M., Khomutov A.R., Janne J., Alhonen L. (2010). Spermidine is indispensable in differentiation of 3t3-l1 fibroblasts to adipocytes. J. Cell. Mol. Med..

[64] Hyvonen M.T., Koponen T., Weisell J., Pietila M., Khomutov A.R., Vepsalainen J., Alhonen L., Keinanen T.A. (2013). Spermidine promotes adipogenesis of 3t3-l1 cells by preventing interaction of anp32 with HUR and pp2a. Biochem. J..

[65] Ishii I., Ikeguchi Y., Mano H., Wada M., Pegg A.E., Shirahata A. (2012). Polyamine metabolism is involved in adipogenesis of 3t3-l1 cells. Amino Acids.

[66] Alhonen L., Rasanen T.L., Sinervirta R., Parkkinen J.J., Korhonen V.P., Pietila M., Janne J. (2002). Polyamines are required for the initiation of rat liver regeneration. Biochem. J..

[67] Oh S.H., Swiderska-Syn M., Jewell M.L., Premont R.T., Diehl A.M. (2018). Liver regeneration requires Yap1-TGFbeta-dependent epithelial-mesenchymal transition in hepatocytes. J. Hepatol..

[68] Lu W.Y., Bird T.G., Boulter L., Tsuchiya A., Cole A.M., Hay T., Guest R.V., Wojtacha D., Man T.Y., Mackinnon A. (2015). Hepatic progenitor cells of biliary origin with liver repopulation capacity. Nat. Cell Biol..

[69] Conigliaro A., Amicone L., Costa V., De Santis Puzzonia M., Mancone C., Sacchetti B., Cicchini C., Garibaldi F., Brenner D.A., Kisseleva T. (2013). Evidence for a common progenitor of epithelial and mesenchymal components of the liver. Cell Death Differ..

[70] Wang B., Zhao L., Fish M., Logan C.Y., Nusse R. (2015). Self-renewing diploid axin2(+) cells fuel homeostatic renewal of the liver. Nature.

[71] Gutierrez E., Shin B.S., Woolstenhulme C.J., Kim J.R., Saini P., Buskirk A.R., Dever T.E. (2013). Eif5a promotes translation of polyproline motifs. Mol. Cell.

[72] Li S.S. (2005). Specificity and versatility of sh3 and other proline-recognition domains: Structural basis and implications for cellular signal transduction. Biochem. J..

[73] Burgio G., Corona D.F., Nicotra C.M., Carruba G., Taibi G. (2016). P/caf-mediated spermidine acetylation regulates histone acetyltransferase activity. J. Enzyme Inhib. Med. Chem..

[74] McCormack S.A., Johnson L.R. (2001). Polyamines and cell migration. J. Physiol. Pharmacol..

[75] Chen C., Young B.A., Coleman C.S., Pegg A.E., Sheppard D. (2004). Spermidine/spermine n1-acetyltransferase specifically binds to the integrin alpha9 subunit cytoplasmic domain and enhances cell migration. J. Cell Biol..

[76] Kohn-Gaone J., Gogoi-Tiwari J., Ramm G.A., Olynyk J.K., Tirnitz-Parker J.E. (2016). The role of liver progenitor cells during liver regeneration, fibrogenesis, and carcinogenesis. Am. J. Physiol..

[77] Yamashita T., Wang X.W. (2013). Cancer stem cells in the development of liver cancer. J. Clin. Investing..

[78] Yamashita T., Ji J., Budhu A., Forgues M., Yang W., Wang H.Y., Jia H., Ye Q., Qin L.X., Wauthier E. (2009). EPCAM-positive hepatocellular carcinoma cells are tumor-initiating cells with stem/progenitor cell features. Gastroenterology.

[79] Ivanov A.V., Valuev-Elliston V.T., Tyurina D.A., Ivanova O.N., Kochetkov S.N., Bartosch B., Isaguliants M.G. (2017). Oxidative stress, a trigger of hepatitis c and b virus-induced liver carcinogenesis. Oncotarget.

[80] Smirnova O.A., Keinanen T.A., Ivanova O.N., Hyvonen M.T., Khomutov A.R., Kochetkov S.N., Bartosch B., Ivanov A.V. (2017). Hepatitis C virus alters metabolism of biogenic polyamines by affecting expression of key enzymes of their metabolism. Biochem. Biophys. Res. Commun..

[81] Gaude E., Frezza C. (2016). Tissue-specific and convergent metabolic transformation of cancer correlates with metastatic potential and patient survival. Nat. Commun..

[82] Liu M., Quek L.E., Sultani G., Turner N. (2016). Epithelial-mesenchymal transition induction is associated with augmented glucose uptake and lactate production in pancreatic ductal adenocarcinoma. Cancer Metab..

