# Supplementary material for: Activation of Polyamine Catabolism by N1,N11-Diethylnorspermine in Hepatic HepaRG Cells Induces Dedifferentiation and Mesenchymal-Like Phenotype
Source: Cells. 2018 Dec 18;7(12):275. doi: 10.3390/cells7120275 (PMC6316793; doi:10.3390/cells7120275)
Supplement: Supplementary file 1 [file cells-07-00275-s001.zip › Supplemental Table S1-S3 Fig S1.docx]

**Supplementary materials for:**

ACTIVATION OF POLYAMINE CATABOLISM BY N^1^,N^11^-DIETHYLNORSPERMINE IN HEPATIC HEPARG CELLS INDUCES DEDIFFERENTIATION AND MESENCHYMAL-LIKE PHENOTYPE

Olga N. Ivanova ^1^, Anastasiya V. Snezhkina ^1^, George S. Krasnov ^1^, Vladimir T. Valuev-Elliston ^1^, Olga A. Khomich ^1,2^, Alexey R. Khomutov ^1^, Tuomo A. Keinanen ^3^, Leena Alhonen ^3^, Birke Bartosch ^2^, Anna V. Kudryavtseva ^1^, Sergey N. Kochetkov ^1^, and Alexander V. Ivanov ^1,^*

^1^ Engelhardt Institute of Molecular Biology, Russian Academy of Sciences, Moscow, Russia; [olgaum@yandex.ru](mailto:olgaum@yandex.ru) (O.N.I.); [leftger@rambler.ru](mailto:leftger@rambler.ru) (A.V.S.); [gskrasnov@mail.ru](mailto:gskrasnov@mail.ru) (G.S.K.); [oakhomich@gmail.com](mailto:oakhomich@gmail.com) (O.A.K.); [alexkhom@list.ru](mailto:alexkhom@list.ru) (A.R.K.); [rhizamoeba@mail.ru](mailto:rhizamoeba@mail.ru) (A.V.K.); [kochet@eimb.ru](mailto:kochet@eimb.ru) (S.N.K.); [aivanov@yandex.ru](mailto:aivanov@yandex.ru) (A.V.I.)

^2^ Cancer Research Center Lyon, INSERM U1052 and CNRS 5286, Lyon University, Lyon, France; [birke.bartosch@inserm.fr](mailto:birke.bartosch@inserm.fr) (B.B.)

^3^ School of Pharmacy, Biocenter Kuopio, University of Eastern Finland, Kuopio, Finland; [tuomo.keinanen@uef.fi](mailto:tuomo.keinanen@uef.fi) (T.K.); [alhonenl@gmail.com](mailto:alhonenl@gmail.com) (L.A.)

***** Correspondence: aivanov@yandex.ru; Tel.: +7-499-135-6065

**Table S1.** Primers used for quantification of gene transcription levels by real-time RT-PCR.

| **Transcript** |  | **Sequence** | **Tm (°C)** | **Amplicon (bp)** |
| --- | --- | --- | --- | --- |
| Albumin | Sense | 5'-TGCTTGAATGTGCTGATGACAGGG-3' | 63.7 | 162 |
|  | Antisense | 5'-AAGGCAAGTCAGCAGGCATCTCATC-3' | 65.4 |  |
| α-Antitrypsin | Sense | 5'-GGGTCAACTGGGCATCACTA-3' | 59.4 | 175 |
|  | Antisense | 5'-GGGGATAGACATGGGTATGG-3' | 56.5 |  |
| CYP 3A4 | Sense | 5'-CCTTACACATACACACCCTTTGGAAGT-3' | 63 | 382 |
|  | Antisense | 5'-AGCTCAATGCATGTACAGAATCCCCGGTTA-3' | 67.7 |  |
| CYP 2C9 | Sense | 5'-CCTCTGGGGCATTATCCATC-3' | 57.5 | 137 |
|  | Antisense | 5'-ATATTTGCACAGTGAAACATAGGA-3' | 56.7 |  |
| Transferrin | Sense | 5'-GTCAACTGTGTCCAGGGTGTGG-3' | 63.3 | 75 |
|  | Antisense | 5'-TCAGACACTTGAAGGCTCCCG-3' | 62.0 |  |
| HNF3β | Sense | 5'-CACCACTACGCCTTCAACCAC-3' | 61.5 | 235 |
|  | Antisense | 5'-GGTAGTAGGAGGTATCTGCGG-3' | 58.8 |  |
| HNF4α | Sense | 5'-CCAAGTACATCCCAGCTTTC-3' | 56.1 | 295 |
|  | Antisense | 5'-TTGGCATCTGGGTCAAAG-3' | 54.8 |  |
| α-Fetoprotein | Sense | 5'-TGCAGCCAAAGTGAAGAGGGAAGA-3' | 64.3 | 217 |
|  | Antisense | 5'-CATAGCGAGCAGCCCAAAGAAGAA-3' | 63.5 |  |
| E-cadherin | Sense | 5'-GAGTGCCAACTGGACCATTC-3' | 58.8 | 82 |
|  | Antisense | 5'-ACCCACCTCTAAGGCCATCT-3' | 60.0 |  |
| N-cadherin | Sense | 5'-TGGAACGCAGTGTACAGAATCAG-3' | 60.9 | 196 |
|  | Antisense | 5'-TTGACTGAGGCGGGTGCTGAATT-3' | 64.8 |  |
| Fibronectin | Sense | 5'-CGAGCTTCCCCAACTGGTAACCC-3' | 65.2 | 526, 433 |
|  | Antisense | 5'-GGTGGCACCTCTGGTGAGGC-3' | 65.2 |  |
| Vimentin | Sense | 5'-AGATGGCCCTTGACATTGAG-3' | 57.6 | 132 |
|  | Antisense | 5'-CCAGAGGGAGTGAATCCAGA-3' | 58.1 |  |
| Snai1 | Sense | 5'-CGAAAGGCCTTCAACTGCAAAT-3' | 60.3 | 262 |
|  | Antisense | 5'-ACTGGTACTTCTTGACATCTG-3' | 55.0 |  |
| Snai2 (Slug) | Sense | 5'-ctgggctggccaaacataag-3' | 59.2 | 78 |
|  | Antisense | 5'-ccttgtcacagtatttacagctgaaag-3' | 60.6 |  |
| Twist | Sense | 5'-GCAAGAAGTCGAGCGAAGAT-3' | 58.4 | 92 |
|  | Antisense | 5'-GCTCTGCAGCTCCTCGAA-3' | 59.4 |  |
| β-actin | Sense | 5'-GATCATTGCTCCTCCTGAGC-3' | 58.1 | 101 |
|  | Antisense | 5'-ACTCCTGCTTGCTGATCCAC-3' | 60.0 |  |
|  | Probe | 5'-[R6G]-CTCGCTGTCCACCTTCCAGCAGAT-[BHQ-1]-3' | 66.5 |  |

**Table S2.** Polyamine levels in the differentiated HepaRG cells treated with DFMO, DENSpm or MDL72.527 for 72 h.

| **Cell line** | **Put^1^** | **Spd^1^** | **Spm^1^** | **N^1^-AcSpd^1^** | **N^8^-AcSpd^1^** | **N^1^-AcSpm^1^** |
| --- | --- | --- | --- | --- | --- | --- |
| Control | 0.85±0.34 | 6.22±2.18 | 8.76±2.10 | N.D.^2^ | N.D. | N.D.^2^ |
| DFMO  (5 mM) | 0.59±0.20 | 4.99±0.92 | 8.85±0.55 | N.D. | N.D. | N.D.^2^ |
| DENSpm  (10 μM) | N.D. | 3.62±0.26 | 2.34±0.08 | N.D. | N.D. | N.D. |
| MDL72.527  (25 μM) | 0.39±0.14 | 3.95±0.94 | 14.49±2.35 | 10.37±4.97 | 59.00±7.47 | 114.97±23.05 |

^1^ (nmol/mg DNA).

^2^ The compound was not detected in all samples.

**Table S2.** Statistical significance between groups, analyzed by Kruskal-Wallis method with Conover-Iman post-hoc approach

|  | **HepaRG^diff^** | **HepaRG^diff^** | **HepaRG^diff^** | **HepaRG^diff^** | **HepaRG^undiff^** | **HepaRG^undiff^** | **HepaRG^undiff^** | **Huh7.5** | **Huh7.5** | **HepG2** |
| --- | --- | --- | --- | --- | --- | --- | --- | --- | --- | --- |
|  | **vs.**  **HepaRG^undiff^** | **vs.**  **Huh7.5** | **vs.**  **HepG2** | **vs.**  **SkHep1** | **vs.**  **Huh7.5** | **vs.**  **HepG2** | **vs.**  **SkHep1** | **vs.**  **HepG2** | **vs.**  **SkHep1** | **vs.**  **SkHep1** |
| AFP | No^1^ | **Yes**^2^ | **Yes**^2^ | **Yes**^2^ | **Yes**^2^ | Tend^3^ | No^1^ | No^1^ | **Yes**^2^ | **Yes**^2^ |
| Alb | **Yes**^2^ | **Yes**^2^ | **Yes**^2^ | **Yes**^2^ | **Yes**^2^ | **Yes**^2^ | **Yes**^2^ | No^1^ | **Yes**^2^ | No^1^ |
| Serpin1 | Tend^4^ | **Yes**^2^ | **Yes**^2^ | **Yes**^2^ | **Yes**^2^ | No^1^ | **Да**^2^ | Tend^4^ | No^1^ | **Yes**^2^ |
| TFR | **Yes**^2^ | No^1^ | No^1^ | **Yes**^2^ | No^1^ | **Yes**^2^ | **Yes**^2^ | No^1^ | **Yes**^2^ | **Yes**^2^ |
| CYP3A4 | **Yes**^2^ | **Yes**^2^ | **Yes**^2^ | **Yes**^2^ | **Yes**^2^ | No^1^ | **Yes**^2^ | No^1^ | No^1^ | **Yes**^2^ |
| CYP2C9 | No^1^ | **Yes**^2^ | **Yes**^2^ | **Yes**^2^ | No^1^ | **Yes**^2^ | **Yes**^2^ | No^1^ | No^1^ | **Yes**^2^ |
| HNF3β | **Yes**^2^ | **Yes**^2^ | No^1^ | **Yes**^2^ | No^1^ | **Yes**^2^ | **Yes**^2^ | No^1^ | **Yes**^2^ | **Yes**^2^ |
| HNF4α | **Yes**^2^ | **Yes**^2^ | **Yes**^2^ | **Yes**^2^ | **Yes**^2^ | No^1^ | **Yes**^2^ | Tend^5^ | No^1^ | **Yes**^2^ |

^1^ p>0,1

^2^ p<0.05

^3^ Tendency to statistical significance, р=0,09

^4^ Tendency to statistical significance, р=0,06

^5^ Tendency to statistical significance, р=0,07





**Figure S1.** DENSpm-treated HepaRG cells do not exhibit elevated ROS production, as revealed with 2’,7’-dichlorodihydrofluoresceine diacetate (DCFHDA). The differentiated HepaRG cells treated with 10 μM DENSpm for 72 h in the absence or presence of 2.5 mM NAC or 100 μM trolox, incubated with 10 μM 2’,7’-dichlorodihydrofluoresceine diacetate (DCFHDA) for 30 min, washed, and fluorescence intensities were recorded on a plate reader with excitation at 485 nm and emission at 535 nm.
