# Supplementary material for: Activation of Polyamine Catabolism by N1,N11-Diethylnorspermine in Hepatic HepaRG Cells Induces Dedifferentiation and Mesenchymal-Like Phenotype
Source: Cells. 2018 Dec 18;7(12):275. doi: 10.3390/cells7120275 (PMC6316793; doi:10.3390/cells7120275)
Supplement: Supplementary file 1 [file cells-07-00275-s001.zip › Table S4. Column annotations.docx]

Gene ID – Ensembl Gene identifier

Symbol – official gene symbol

Biotype – gene type

Name – gene name

RefSeq_Summary – summary (from Reference Sequence)

Annotation – Gene Ontology, KEGG, Reactome annotations

LogFC – binary logarithm of expression fold change

LogCPM – binary logarithm of average read count per million (CPM)

p (QLF test) – p-value (quasi-likelihood F-test)

FDR (QLF test) – false discovery rate (quasi-likelihood F-test)

p (t-test) – p-value (Student’s t-test)

CPMs - read counts per million

 Sparklines (columns H, I) demonstrate the binary logarithms of ratio (CPM each replicate) / (average CPM), vertical scale from -2 to +2, e.g. from 4-fold decrease to 4-fold increase (relatively the average CPM)
